# Impact of hormonal treatment duration in combination with radiotherapy for locally advanced prostate cancer: Meta-analysis of randomized trials

**DOI:** 10.1186/1471-2407-10-675

**Published:** 2010-12-09

**Authors:** Federica Cuppone, Emilio Bria, Diana Giannarelli, Vanja Vaccaro, Michele Milella, Cecilia Nisticò, Enzo Maria Ruggeri, Isabella Sperduti, Sergio Bracarda, Paola Pinnarò, Gaetano Lanzetta, Paola Muti, Francesco Cognetti, Paolo Carlini

**Affiliations:** 1 Department of Medical Oncology, Regina Elena National Cancer Institute, Roma, Italy; 2 Biostatistics, Regina Elena National Cancer Institute, Roma, Italy; 3 Medical Oncology, Ospedale 'Belcolle', Viterbo, Italy; 4 Clinical Oncology Unit, Istituto Neurotraumatologico Italiano (I.N.I.), Grottaferrata, Rome, Italy; 5 Scientiphic Direction, Regina Elena National Cancer Institute, Roma, Italy; 6 Medical Oncology, Ospedale San Donato, Arezzo, Italy

## Abstract

**Background:**

Hormone therapy plus radiotherapy significantly decreases recurrences and mortality of patients affected by locally advanced prostate cancer. In order to determine if difference exists according to the hormonal treatment duration, a literature-based meta-analysis was performed.

**Methods:**

Relative risks (RR) were derived through a random-effect model. Differences in primary (biochemical failure, BF; cancer-specific survival, CSS), and secondary outcomes (overall survival, OS; local or distant recurrence, LR/DM) were explored. Absolute differences (AD) and the number needed to treat (NNT) were calculated. Heterogeneity, a meta-regression for clinic-pathological predictors and a correlation test for surrogates were conducted.

**Results:**

Five trials (3,424 patients) were included. Patient population ranged from 267 to 1,521 patients. The longer hormonal treatment significantly improves BF (with significant heterogeneity) with an absolute benefit of 10.1%, and a non significant trend in CSS. With regard to secondary end-points, the longer hormonal treatment significantly decrease both the LR and the DM with an absolute difference of 11.7% and 11.5%. Any significant difference in OS was observed. None of the three identified clinico-pathological predictors (median PSA, range 9.5-20.35, Gleason score 7-10, 27-55% patients/trial, and T3-4, 13-77% patients/trial), did significantly affect outcomes. At the meta-regression analysis a significant correlation between the overall treatment benefit in BF, CSS, OS, LR and DM, and the length of the treatment was found (p≤0.03).

**Conclusions:**

Although with significant heterogeneity (reflecting different patient' risk stratifications), a longer hormonal treatment duration significantly decreases biochemical, local and distant recurrences, with a trend for longer cancer specific survival.

## Background

Androgen-deprivation remains the cornerstone of treatment for patients with hormone-sensitive advanced prostate cancer [1]. The combination of hormonal suppression and the radiotherapy is able to significantly decrease the recurrences and the mortality of patients affected by locally advanced prostate cancer. Substantial toxicities were also identified when hormone therapy was used.[2-11]. A recently conducted meta-analysis has demonstrated an overall absolute benefit of 7.5-10% in favor of the addition of the hormone therapy to in terms of biochemical failure and clinical progression free survival [12].

The optimal duration of androgen ablation therapy is still controversial despite a growing number of prognostic factors that have been shown to predict disease outcome [13]. In all prospective randomized trials, long term hormonal therapy (more than 2 years) improves survival in patients with locally high risk (Gleason score ≥ 8, median PSA ≥ 20, stage ≥ T3) advanced prostate cancer. This strategy, however, is also associated with an increased risk of morbidities, such as osteoporosis, diabetes, depression, dislipidemia and abdominal obesity [14,15].

The value of short-term hormonal treatment in patients with intermediate risk disease (Gleason score ≥ 7, median PSA ≥ 10, stage ≥ T2c) has also been detected [16,17], although its role in high-risk disease is still controversial. Nevertheless, short courses of hormonal suppression have been shown to be very effective in downstaging localized disease. Surgical series have provided some histopathological confirmation, with significant decreases in margin-positive rates in prostatectomy after neoadjuvant hormonal therapy [18,19].

In order to determine if difference exists between a shorter and a longer hormone therapy (HT) in combination with radiotherapy, a literature based meta-analysis was conducted.

## Methods

The analysis was conducted following 4 steps: definition of the outcomes (definition of the question the analysis was designed to answer), definition of the trial selection criteria, definition of the search strategy, and a detailed description of the statistical methods used [20,21].

### Outcome definition

The combination of a short-term HT (ST) and RT was considered as the experimental arm and a long-term HT (LT) and RT as the standard comparator. Analysis was conducted in order to find significant differences in primary and secondary outcomes, according to a previously published meta-analysis and the reported sequence and definitions in the selected trials. Primary outcomes for the magnitude of the benefit analysis were both the Biochemical Failure (BF, time between randomization and PSA increase) and the CSS (time between randomization and death for prostate cancer). Secondary end-points were: OS (time between randomization and death for any cause), local failure rate (LF) and distant metastases rate (DM).

### Search strategy

Deadline for trial publication and/or presentation was September 30^th^, 2010. Updates of Randomized Clinical Trials (RCTs) were gathered through Medline (PubMed: http://www.ncbi.nlm.nih.gov/PubMed), ASCO (American Society of Clinical Oncology, http://www.asco.org), ESMO (European Society for Medical Oncology, http://www.esmo.org), FECS (Federation of European Cancer Societies, http://www.fecs.be), and ASTRO (American Society for Therapeutic Radiology and Oncology, http://www.astro.org) website searches. Key-words used for searching were: adjuvant hormone therapy, prostate cancer, radiotherapy, duration, longer, shorter, review, metanalysis, meta-analysis, pooled analysis, randomized, phase III, comprehensive review, systematic review. In addition to computer browsing, review and original papers were also scanned in the reference section to look for missing trials. Furthermore, lectures at major meetings (ASCO, ESMO, ECCO, and ASTRO) having 'hormone treatment and radiotherapy for prostate cancer' as the topic were checked. No language restrictions were applied.

### Trial identification criteria

All prospective phase III RCTs published in peer-reviewed journals or presented at the ASCO, ECCO, ESMO and ASTRO meetings until September 2010, in which previously untreated patients with locally advanced prostate cancer were prospectively randomized to short or long-HT plus radiotherapy were gathered. Any trial exploring a shorter versus a longer HT (regardless of their absolute values) in combination with RT for the treatment of LAPC was considered eligible.

### Data extraction

The number of events for primary and secondary end-points were extracted; the last trial's available update was considered as the original source. All data were reviewed and separately computed by five investigators (F.Cu., E.B., D.G., I.S., and P.C.).

### Data synthesis

The log of relative risk ratio (RR) was estimated for each considered endpoint [22], and 95% Confidence Intervals (CI) were derived [23]. A random-effect model according to the DerSimonian method was preferred to the fixed, given the known clinical heterogeneity of trials [24-26]; a Q-statistic heterogeneity test was used [27-29]. Absolute benefits for each outcome were calculated (i.e. absolute benefit=exp {RR × log[control survival]} - control survival [30]; modified by Parmar and Machin [31]). The number of patients needed to be treated for one single beneficial patient was determined (NNT: 1/[(Absolute Benefit)/100]) [32]. Results were depicted in all figures as conventional meta-analysis forest plots; a RR <1.0 indicates fewer events in the experimental arm. In order to find possible correlations between outcome effect and negative prognostic factors (selected among trials' reported factors: the median PSA, the Gleason score of 7-10 and the T3-4), a meta-regression approach was adopted (i.e. regression of the selected predictor on the Log RR of the corresponding outcome); the time delay in months for each considered trial between the duration of the short and the long treatment arm was considered in the meta-regression analysis as treatment predictor as well. Calculations were accomplished using the Comprehensive Meta-Analysis Software, version v. 2.0 (CMA, Biostat, Englewood, NJ, USA).

### Correlation

Potential correlations to test surrogacy between primary and secondary end-points, were explored according to regression between the calculated RRs and their logs for each outcome.

## Results

### Selected trials

**Figure 1:**
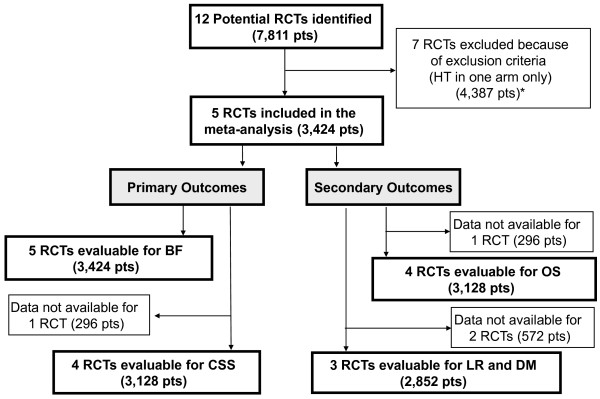
**Outline of the search - Flow diagram**. RCTs: randomized clinical trials; pts: patients; HT: hormone therapy; BF: biochemical failure; OS: overall survival; CSS: cancer specific survival; LR: local recurrences; DM: distant metastases.

Twelve trials (7,811 patients) were identified (Figure 1) [2-11,33-37]. Seven RCTs were excluded because HT was administered in one arm only (4,387 patients) [2-11,33], question already answered in a previous meta-analysis [38]. Five RCTs were included in the meta-analysis, all evaluable for BF (3,424 patients) [7,34-37]. According to the trial' selection, the shorter approach ranged from 3 to 6 months, while the longer from 8 to 36 months; trials characteristics are listed in Table 1. Four were evaluable for CSS, OS, and three for LR and DM [34-37]. Toxicity analysis was not performed because data about toxicity were available only in two studies. Median follow-up ranged from 3.7 to 10 years. Three predictors were identified: median PSA (range 9.5-20.35), Gleason score 7-10 (range 27-55% of patients/trial) and T3-4 (range 13-77% patients/trial).

**Table 1:** Trials' Characteristics.

Authors	Pts	Dose RT (Gy)	Arms (mo.)	Median **F.U**.	End-Points		Median PSA (ng/mL)	N° of pts (%)	
							
					Primary	Secondary		Gleason Score 7-10	T3-T4
*Laverdiere et al*	148 148	64	5 (AS) 10 (AS)	3.7 yrs	BDFS, CDFS	-	11.9 12.7	82 (27.6)	40 (13.4)

*Crook et al*	177 184	66	3 (GF) 8 (GF)	79 mo.	OS, CSS, DFS	-	8.9 10.1	178 (49.3)	48 (13.2)

*Horwitz et al*	763 758	65-70	4 (GF) 24 (GF)	10 yrs	DFS	OS, BF, DMF	20.8 19.8	838 (55.1)	829 (54.5)

*Bolla et al*	483 487	70	6 (CAB) 36 (CAB)	5.2 yrs	OS, CPFS, DS, BPFS	-	18.8 18.8	479 (49.3)	754 (77.7)

*Armstrong et al*	127 134	50-74	4 (CAB) 8 (CAB)	102 mo.	BF	OS, CSS	17.0 13.6	153 (55.4)	159 (57.6)

Pts: patients; RT: radiotherapy; Gy: gray; mo.: months; F.U.: follow-up; PSA: serum prostate-specific antigen; N°: number; AS: androgen suppression; yrs: years; BDFS: biochemical disease free survival; CDFS: clinical disease free survival; GF: goserelin + flutamide; OS: overall survival; CSS: cancer specific survival; DFS: disease free survival; BF: biochemical failure; DMF: distant metastases failure; CAB: complete androgen blockade; CPFS: clinical progression free survival; BPFS: biochemical progression free survival: BF: biochemical failure.

### Combined Analysis

With regard to the primary outcomes, the longer HT significantly decreased biochemical failure over the shorter HT with an absolute benefit of 10.1% (RR 1.32, 95% CI 1.09, 1.60, p = 0.004), corresponding to 9-10 patients to be treated for one to benefit, although with significant heterogeneity (p = 0.003) (Table 2). A non significant trend in the prostate-cancer specific survival was found (RR 1.21, 95% CI 0.82, 1.79, p = 0.32) when adopting a longer HT, without significant heterogeneity. Concerning the secondary outcomes, the longer HT significantly reduced both the risk of local recurrences (RR 1.87, 95% CI 1.22, 2.86, p = 0.004) and the risk of distant metastases (RR 1.77, 95% CI 1.16, 2.69, p = 0.007) by 11.7% and 11.5%, without significant heterogeneity, which translate into 9 patients to be treated for one to benefit (Table 2). No significant differences in overall survival were observed by comparing the two arms (Table 2).

**Table 2:** Combined efficacy results.

Outcomes	Pts (RCTs)	RR (95% CI)	*p-value*	Het. (*p*)	AD (%)	NNT
BF	3,424 (5)	1.32 (1.09, 1.60)	*0.004*	*0.003*	10.1	9-10

CSS	3,128 (4)	1.21 (0.82, 1.79)	*0.32*	*0.09*	-	-

OS	3,128 (4)	1.09 (0.92, 1.28)	*0.28*	*0.15*	-	-

DM	2,852 (3)	1.77 (1.16, 2.69)	*0.007*	*0.06*	11.5	9

LR	2,852 (3)	1.87 (1.22, 2.86)	*0.004*	*0.10*	11.7	9

Pts: patients; RCTs: randomized clinical trials; RR: relative risk; CI: confidence intervals; Het.: heterogeneity; p: p-value; AD: absolute difference; NNT: number needed to treat; BF: biochemical failure; CSS: cancer specific survival; OS: overall survival: LR: local relapse; DM: distant metastases.

According to the performed meta-regression analysis, none of the considered negative predictors significantly affected outcome. Conversely, the treatment predictor (the time delay) significantly correlates with all explored outcomes (Table 3).

**Table 3:** Meta-regression Analysis.

Outcome	Predictor p-value			
**Log RR**	**Median PSA**	**G 7-10**	**T3-4**	**Time Delay**

BF	*0.34*	*0.35*	*0.43*	***0.0003***

CSS	*0.11*	*0.81*	*0.19*	***0.03***

OS	*0.50*	*0.15*	*0.06*	***0.03***

LR	*0.21*	*0.70*	*0.18*	***0.03***

DM	*0.81*	*0.23*	*0.63*	***0.02***

RR: relative risk; G: Gleason score; T: t-size; Time delay: time interval between the short and the long hormonal treatment duration; BF: biochemical failure; CSS: cancer specific survival; OS: overall survival: LR: local relapse; DM: distant metastases.

### Correlation Analysis

The correlation analysis was performed in the 4 RCTs, in which BF could be considered as a potential surrogate endpoint [34-36]. The regression between the RR of BF and the Log of CSS (p = 0.005) was statistically significant. The regression between the RR of OS and the Log of BF and CSS was not significant (p = 0.052 and p = 0.16, respectively). The regression of the RR of DM and the Log of BF and CSS was statistically significant (p = 0.029 and (p = 0.041, respectively). The regression of the RR of LR and the Log of BF was statistically significant (p = 0.049).

## Discussion

**Figure 2:**
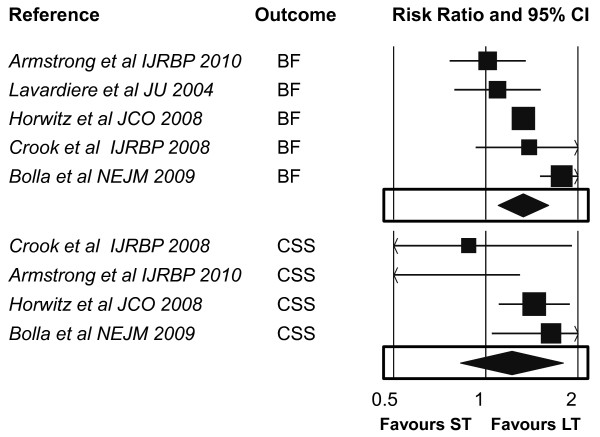
**Combined Results - Primary Outcomes (BF, CSS)**. CI: confidence intervals; BF: biochemical failure; CSS: cancer specific survival; ST: shorter therapy: LT: longer therapy.

**Figure 3:**
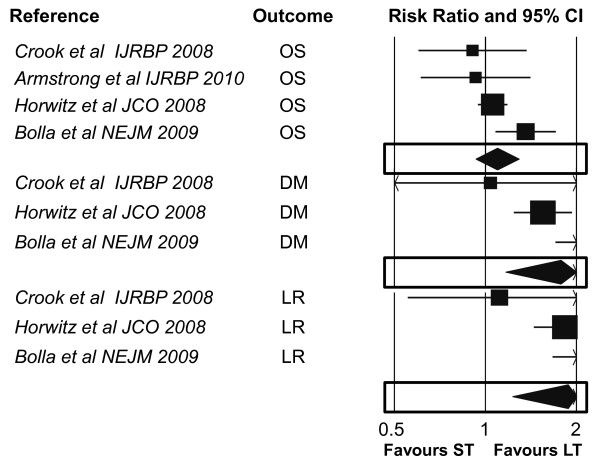
**Combined Results - Secondary Outcomes (OS, DM, LR)**. CI: confidence intervals; OS: overall survival; DM: distant metastases; LR: local recurrences; ST: shorter therapy: LT: longer therapy.

Although the number of studies is small to produce reliable estimates, the data presented herein strengthen the role of the hormone-therapy duration in patients candidates to receive androgen suppression in association with radiotherapy for locally advanced prostate cancer (Table 2 Figures 2, 3).

Hormonal suppression in patients affected by locally advanced prostate cancer candidates to receive exclusive radiotherapy is able to improve outcome, as a series of trials recently demonstrated; according to the results of our previous meta-analysis, hormonal suppression plus radiotherapy significantly decreases recurrence and mortality of patients with localized prostate cancer. The magnitude of this survival benefit ranges from 4.9% to 5.5% for OS and CSS, respectively, which translates into 20 and 18 NNT. Local and distant relapse were significantly decreased by hormonal treatment, by 36% and 28%, without significantly affect toxicity [38].

Although the use of adjuvant hormonal therapy has been shown in randomized trials to improve outcomes, two issues still need to be clarified: 1) the optimal hormone therapy duration and 2) the impact of the radiation dose on outcome and local control. Within this context, the current meta-analysis was aimed to assess if difference exist between a shorter and longer androgen suppression, according to the trialist' definition. The pooled results of the considered trials show that a longer treatment approach improves all outcomes when compared to a shorter one (Table 2 Figure 2, 3). In particular, BF is significantly reduced when hormone-therapy is administered in a longer fashion, with an overall absolute difference of 10.1%, which corresponds to only 9-10 patients needed to be treated for one to benefit (Table 2). Although the meta-regression analysis could be not valid given the small number of gathered trials, the benefits present across all outcomes are provided in favour of a longer strategy, regardless of any of the selected negative predictors, suggesting an overall effect independent by any clinico-pathological feature. Conversely to the these predictors, the lenght of treatment seems to significantly affect all outcomes, suggesting the even more independent value of a longer approach (Table 3). Although this interpretation should be softened, given the limitations related to a meta-regression analysis, a further individual patient data meta-analysis could eventually clarify if a particular subset of patients would better benefit of such approach. Moreover, these conclusion should be driven only when an homogenous risk classes classification was likely to be prospectively adopted in all analyzed trials. Indeed, the significant heterogeneity in the selected primary outcome can easily reflect that the magnitude of the reported benefit is different across trials, and this can likely be a clear effect of the different patient' selection.

The adopted meta-analytic method did not allow to extract patients' subgroups data from those trials where either low, intermediate and high risk patients were accrued together. For example, no advantage for longer duration was found in those trials enrolling low risk patients [7,39] (Figure 2, 3). Conversely, the positive effect of long term hormonal duration can be partially driven by the inclusion of those 2 trials including patients with node-positive disease [34,36]. So, we are not able to actually exclude a 'carry-over' effect due to the inclusion of such trials with these characteristics. The mixture of such confounders (such as different cut-offs in the Gleason score) in trial selection does not allow to derive clear and definitive conclusions.

Nevertheless, even taking into account all the drawbacks in correlation and meta-regression estimations of cumulating few trials, the absolute difference in favour of the longer HT (and the lower NNT) strongly justifies a prolonged treatment strategy. Moreover, the meta-regression analysis did also show a significant correlation between the length of the longer strategy and the effect in the reduction of BF, LR and DM and the improvement of CSS (Table 3 Figure 2). The latest conclusion allows to carefully speculate upon a 'treat-until-progression' strategy, at least in the context of a randomized trial.

Recent reports suggest that the risk of cardio metabolic problems with long-term castration deprivation therapy could counteract the benefits of hormone therapy, although this has also been questioned. Long term androgen suppression can reduce the quality of life and increase the risk of fatal myocardial infarction, fractures, and the development of a metabolic syndrome. In the context of neoadjuvant approaches, it has been recently reported that hormone-therapy is associated with a significant increase of all-cause mortality in those patients with a cardiac comorbidity, but not in those who did not show any history of cardiovascular disease [40]. This represent a crucial step-forward in the selection of those patient who could be considered as appropriate candidates for a longer hormonal suppression.

The radiotherapy component of the combined treatment is crucial: cancer-specific and overall mortality rates at 10 years are significantly lower with the combined treatment in respect to androgen suppression alone. Moreover, the survival benefit seem to depend on the duration of homone-therapy, and was also reported by the Early Prostate Cancer Programme using adjuvant antiandrogen treatment as well. In studies with short or intermediate androgen deprivation of, survival prolongation has only been reported in subgroups.

At the start of these trials, the standard radiation dose to the prostate was 70 Gy or less. With the advent of intensity-modulated and image-guided radiotherapy techniques, radiation doses of 78 Gy or higher are now possible, and randomised studies have shown that biochemical relapse-free survival improves with high radiation doses. As a consequence, the overall survival benefit might further increase further with higher safely radiation doses [41-44]. Clearly, open issues remain whether the duration of the hormonal suppression could eventually affect the outcome when radiation therapy is administered with higher dosages, and what should be irradiated (prostate or the whole pelvis).

The general shared consensus is that high risk patients would better benefit from two to three years of adjuvant androgen deprivation, while those with intermediate' risk prostate cancer from six months of adjuvant androgen deprivation plus radiotherapy delivered with a dose lower or equal to 70Gy (for doses higher than 70 Gy the appropriate duration of adjuvant androgen deprivation is still unknown) [16,17]. According to the trial recently published by Bolla et al, the effect of short-term and long-term androgen suppression upon five-year mortality is likely to be maintained at ten years [39,45], whereas the short-term benefit may actually be no longer effective. The intriguing factor that can help in the daily decision between one strategy over the other is the relative weight of adverse events and the adverse effects on quality of life between the two groups of patients; indeed, although no significant difference in the overall quality of life did emerge between the two groups in the Bolla et al trial (p = 0.37), a clinically meaningful difference was present for hot flushes, sexual interest, and sexual activity [34]. Unfortunately, our analysis could investigate neither the pooled toxicities nor the quality of life data into a cumulative fashion, given the lack of a complete reporting in the original trials.

Although the treatment duration seems to significantly impact upon outcome, it should be acknowledged that other factors may have a critical role, or may be more important: indeed, in the recent update of the Crook et al study, the more relevant weight for benefit seems related more to the early biochemical response to hormonal treatment before radiotherapy, rather than its overall duration [46]. For these reasons, a critical view of all data as shown in our analysis (taking into account all the limitations related to the adopted approach and methodology) is recommended.

## Conclusions

Taking into account the current data available in literature, and the results reported herein, the significant differences in outcome in favour of a longer hormonal treatment duration do support the adoption of such a strategy for patients affected by locally advanced prostate cancer, although several limitations should be considered given the small number of trials included. Nevertheless, a careful patient selection upon the presence/absence of cardiovascular and/or metabolic comorbidities should be adopted, in order to avoid significan late-term toxicities. On the contrary, a wide adoption of a longer administration regardless of the risk stratification (notwithstanding the results of the meta-regression analysis) seems in some way not enough mature, in absence of more reliable data coming from an individual patient data meta-analysis.

## Competing interests

None of the authors have competing interests or potential conflicts with this work. In particular:

• In the past five years have you received reimbursements, fees, funding, or salary from an organization that may in any way gain or lose financially from the publication of this manuscript, either now or in the future? NO

• Do you hold any stocks or shares in an organization that may in any way gain or lose financially from the publication of this manuscript, either now or in the future? NO

• Do you hold or are you currently applying for any patents relating to the content of the manuscript? Have you received reimbursements, fees, funding, or salary from an organization that holds or has applied for patents relating to the content of the manuscript? NO

• Do you have any other financial competing interests? NO

## Authors' contributions section

FCu, EB, DG, and PC conceived the analysis, and supervised the calculations; FCu, EB, DG, IS, and PC performed the calculations in a blinded fashion; VV, MM, EMR, IS, and PC participated in the trials recruitment and selection process; FCu, EB, SB and PC drafted and revised the manuscript; DG, CN, PP, GL, PM, FC and PC did coordinate the overall study process and did provide the funding. All authors read and approved the final manuscript.

## Pre-publication history

The pre-publication history for this paper can be accessed here:

http://www.biomedcentral.com/1471-2407/10/675/prepub
